# The genome sequence of a parasitoid wasp, *Netelia inedita* (Kokujev, 1899) (Hymenoptera: Ichneumonidae)

**DOI:** 10.12688/wellcomeopenres.26144.1

**Published:** 2026-03-18

**Authors:** Gavin R. Broad, Natural History Museum Genome Acquisition Lab, Darwin Tree of Life Barcoding Collective, Wellcome Sanger Institute Tree of Life Management, Samples and Laboratory team, Wellcome Sanger Institute Scientific Operations: Sequencing Operations, Wellcome Sanger Institute Tree of Life Core Informatics team, Tree of Life Core Informatics collective, Darwin Tree of Life Consortium

**Affiliations:** 1 Natural History Museum, London, England, UK

**Keywords:** Netelia inedita; parasitoid wasp; genome sequence; chromosomal; Hymenoptera

## Abstract

We present a genome assembly from an individual female *Netelia inedita* (parasitoid wasp; Arthropoda; Insecta; Hymenoptera; Ichneumonidae). The genome sequence has a total length of 403.61 megabases. Most of the assembly (56.53%) is scaffolded into 6 chromosomal pseudomolecules. The mitochondrial genome has also been assembled, with a length of 26.77 kilobases. This assembly was generated as part of the Darwin Tree of Life project, which produces reference genomes for eukaryotic species found in Britain and Ireland.

## Species taxonomy

Eukaryota; Opisthokonta; Metazoa; Eumetazoa; Bilateria; Protostomia; Ecdysozoa; Panarthropoda; Arthropoda; Mandibulata; Pancrustacea; Hexapoda; Insecta; Dicondylia; Pterygota; Neoptera; Endopterygota; Hymenoptera; Apocrita; Ichneumonoidea; Ichneumonidae; Tryphoninae; Phytodietini; *Netelia*; *Paropheltes*; *Netelia inedita* (Kokujev, 1899) (NCBI:txid3458737).

## Background

*Netelia inedita* is a widespread and sometimes common species of parasitoid wasp and is fairly easily distinguished from other *Netelia* species. Unfortunately, that hasn’t prevented the wrong names being used for this species pretty consistently throughout the literature. *Paniscus ineditus* Kokujev, 1899, is listed as a junior synonym of *Netelia thomsonii* (Brauns, 1889) in most sources, following Tolkanitz (1974) and Kasparyan & Tolkanitz (1999). However, *Netelia inedita* and *N. thomsonii* are clearly separate species, as recognised by Delrio (1974). Broad *et al.* (in prep.), in a review of European *Netelia*, separate the two species. *Netelia inedita* belongs to the subgenus *Paropheltes*, with no lateral carinae on the scutellum and pale creamy white markings on the mesosoma (more conspicuous in males, nearly absent in females). Within *N.* ( *Paropheltes*), *N. inedita* can be identified by the distinctive male genitalia (the gonostyle has a triangularly widened brace across the inner surface and a small, striate pad) and fore wing vein 1 *cu-a* separated from *M&RS* by about 0.3 of the length of 1 *cu-a.* Compared to *N. thomsonii*, the mesoscutum is more roughly sculptured and the pale marks weaker. The male of *N. thomsonii* has a small tooth on the margin of the gonostyle. In older collections, *N. inedita* was often identified as ‘ *Paniscus longipes*’ or ‘ *Paniscus gracilipes*’, both names now recognised as junior synonyms of other *Netelia* species.

As with other *Netelia* species, *N. inedita* is primarily nocturnal and a koinobiont ectoparasitoid of Lepidoptera larvae. Shaw (2001) gives some information on the biology and Broad *et al.* (in prep.) summarize the ecology of *N. inedita.* Most host records are from *Xanthorhoe fluctuata* (Linnaeus) (Garden Carpet) but it attacks other Geometridae hosts of the subfamily Larentiinae, such as *Lithostege griseata* (Denis & Schiffermüller) (Grey Carpet). The host is stung as a fully grown larva and temporarily paralysed while the dark, hard egg is anchored into the cuticle. When the host is preparing its pupation site, the *N. inedita* larva rapidly consumes it and then spins its tough, black cocoon. There are at least two generations per year, with adults on the wing in late spring and early summer then again later in the summer and autumn, being particularly frequent in the autumn. Found throughout the UK, *N. inedita* has a wide range across Europe (Broad *et al.*, in prep.) although published data are difficult to interpret because of the frequent misidentifications.

This is the first genome sequence for a species of the subgenus *N.* ( *Paropheltes*), joining several other Darwin Tree of Life genomes of *Netelia* species, published ( Broad *et al.*, 2024; Broad *et al.*, 2025; Price *et al.*, 2024) and presently in assembly, which are enabling population genetics work on a group of parasitoids for which distribution and ecological data are comparatively well-known.

## Methods

### Sample acquisition and DNA barcoding

**Figure 1.**
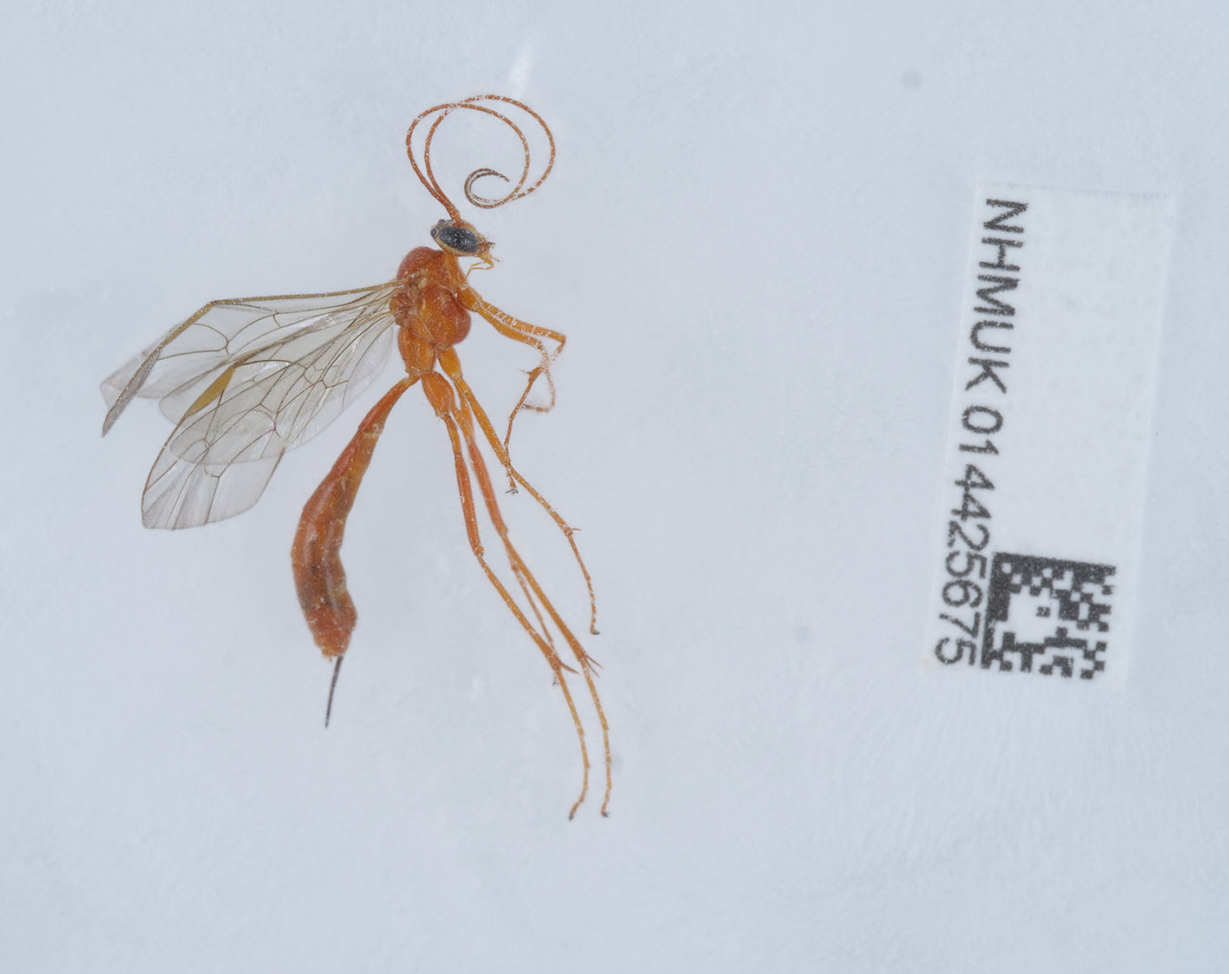
Photograph of the *Netelia inedita* (iyNetThom1) specimen used for genome sequencing.

The specimen used for genome sequencing was an adult female *Netelia inedita* (specimen ID NHMUK014425675, ToLID iyNetThom1; Figure 1), collected from Tonbridge, England, United Kingdom (latitude 51.19, longitude 0.27) on 2021-06-25. The specimen was collected and identified by Gavin Broad (Natural History Museum). The same specimen was used for RNA sequencing.

The initial identification was verified by an additional DNA barcoding process according to the framework developed by Twyford *et al.* (2024). A small sample was dissected from the specimen and stored in ethanol, while the remaining parts were shipped on dry ice to the Wellcome Sanger Institute (WSI) (see the protocol). The tissue was lysed, the COI marker region was amplified by PCR, and amplicons were sequenced and compared to the BOLD database, confirming the species identification ( Crowley *et al.*, 2023). Following whole genome sequence generation, the relevant DNA barcode region was also used alongside the initial barcoding data for sample tracking at the WSI ( Twyford *et al.*, 2024). The standard operating procedures for Darwin Tree of Life barcoding are available on protocols.io.

### Nucleic acid extraction

Protocols for high molecular weight (HMW) DNA extraction developed at the Wellcome Sanger Institute (WSI) Tree of Life Core Laboratory are available on protocols.io ( Howard *et al.*, 2025). The iyNetThom1 sample was weighed and triaged to determine the appropriate extraction protocol. Tissue from the thorax was homogenised by powermashing using a PowerMasher II tissue disruptor.

HMW DNA was extracted in the WSI Scientific Operations core using the Automated MagAttract v2 protocol. Sheared DNA was purified by manual SPRI (solid-phase reversible immobilisation). The concentration of the sheared and purified DNA was assessed using a Nanodrop spectrophotometer and Qubit Fluorometer using the Qubit dsDNA High Sensitivity Assay kit. Fragment size distribution was evaluated by running the sample on the FemtoPulse system. For this sample, the final post-shearing DNA had a Qubit concentration of 16.0 ng/μL and a yield of 736.00 ng, with a fragment size of 15.2 kb.

RNA was extracted from abdomen tissue of iyNetThom1 in the Tree of Life Laboratory at the WSI using the RNA Extraction: Automated MagMax™ *mir*Vana protocol. The RNA concentration was assessed using a Nanodrop spectrophotometer and a Qubit Fluorometer using the Qubit RNA Broad-Range Assay kit. Analysis of the integrity of the RNA was done using the Agilent RNA 6000 Pico Kit and Eukaryotic Total RNA assay.

### PacBio HiFi library preparation and sequencing

Library preparation and sequencing were performed at the WSI Scientific Operations core. Libraries were prepared using the SMRTbell Prep Kit 3.0 (Pacific Biosciences, California, USA), following the manufacturer’s instructions. The kit includes reagents for end repair/A-tailing, adapter ligation, post-ligation SMRTbell bead clean-up, and nuclease treatment. Size selection and clean-up were performed using diluted AMPure PB beads (Pacific Biosciences). DNA concentration was quantified using a Qubit Fluorometer v4.0 (ThermoFisher Scientific) and the Qubit 1X dsDNA HS assay kit. Final library fragment size was assessed with the Agilent Femto Pulse Automated Pulsed Field CE Instrument (Agilent Technologies) using the gDNA 55 kb BAC analysis kit.

The sample was sequenced using the Sequel IIe system (Pacific Biosciences, California, USA). The concentration of the library loaded onto the Sequel IIe was in the range 40–135 pM. The SMRT link software, a PacBio web-based end-to-end workflow manager, was used to set-up and monitor the run, and to perform primary and secondary analysis of the data upon completion.

### Hi-C

***Sample preparation and crosslinking***

The Hi-C sample was prepared from 20–50 mg of frozen head tissue from the iyNetThom1 sample using the Arima-HiC v2 kit (Arima Genomics). Following the manufacturer’s instructions, tissue was fixed and DNA crosslinked using TC buffer to a final formaldehyde concentration of 2%. The tissue was homogenised using the Diagnocine Power Masher-II. Crosslinked DNA was digested with a restriction enzyme master mix, biotinylated, and ligated. Clean-up was performed with SPRISelect beads before library preparation. DNA concentration was measured with the Qubit Fluorometer (Thermo Fisher Scientific) and Qubit HS Assay Kit. The biotinylation percentage was estimated using the Arima-HiC v2 QC beads.

***Hi-C library preparation and sequencing***

Biotinylated DNA constructs were fragmented using a Covaris E220 sonicator and size selected to 400–600 bp using SPRISelect beads. DNA was enriched with Arima-HiC v2 kit Enrichment beads. End repair, A-tailing, and adapter ligation were carried out with the NEBNext Ultra II DNA Library Prep Kit (New England Biolabs), following a modified protocol where library preparation occurs while DNA remains bound to the Enrichment beads. Library amplification was performed using KAPA HiFi HotStart mix and a custom Unique Dual Index (UDI) barcode set (Integrated DNA Technologies). Depending on sample concentration and biotinylation percentage determined at the crosslinking stage, libraries were amplified with 10–16 PCR cycles. Post-PCR clean-up was performed with SPRISelect beads. Libraries were quantified using the AccuClear Ultra High Sensitivity dsDNA Standards Assay Kit (Biotium) and a FLUOstar Omega plate reader (BMG Labtech).

Prior to sequencing, libraries were normalised to 10 ng/μL. Normalised libraries were quantified again to create equimolar and/or weighted 2.8 nM pools. Pool concentrations were checked using the Agilent 4200 TapeStation (Agilent) with High Sensitivity D500 reagents before sequencing. Sequencing was performed using paired-end 150 bp reads on the Illumina NovaSeq 6000.

### RNA library preparation and sequencing

Libraries were prepared using the NEBNext ^®^ Ultra™ II Directional RNA Library Prep Kit for Illumina (New England Biolabs), following the manufacturer’s instructions. Poly(A) mRNA in the total RNA solution was isolated using oligo (dT) beads, converted to cDNA, and uniquely indexed; 14 PCR cycles were performed. Libraries were size-selected to produce fragments between 100–300 bp. Libraries were quantified, normalised, pooled to a final concentration of 2.8 nM, and diluted to 150 pM for loading. Sequencing was carried out on the Illumina NovaSeq 6000, generating paired-end reads.

### Genome assembly

Prior to assembly of the PacBio HiFi reads, a database of *k*-mer counts ( *k* = 31) was generated from the filtered reads using FastK. GenomeScope2 ( Ranallo-Benavidez *et al.*, 2020) was used to analyse the *k*-mer frequency distributions, providing estimates of genome size, heterozygosity, and repeat content.

The HiFi reads were assembled using Hifiasm ( Cheng *et al.*, 2021) with the --primary option. Haplotypic duplications were identified and removed using purge_dups ( Guan *et al.*, 2020). The Hi-C reads ( Rao *et al.*, 2014) were mapped to the primary contigs using bwa-mem2 ( Vasimuddin *et al.*, 2019), and the contigs were scaffolded in YaHS ( Zhou *et al.*, 2023) with the --break option for handling potential misassemblies. The scaffolded assemblies were evaluated using Gfastats ( Formenti *et al.*, 2022), BUSCO ( Manni *et al.*, 2021) and MERQURY.FK ( Rhie *et al.*, 2020).

The mitochondrial genome was assembled using MitoHiFi ( Uliano-Silva *et al.*, 2023).

### Assembly curation

The assembly was decontaminated using the Assembly Screen for Cobionts and Contaminants ( ASCC) pipeline. TreeVal was used to generate the flat files and maps for use in curation. Manual curation was conducted primarily in PretextView and HiGlass ( Kerpedjiev *et al.*, 2018). Scaffolds were visually inspected and corrected as described by Howe *et al*. (2021). Manual corrections included 24 breaks, 44 joins, and removal of two haplotypic duplications. This reduced the scaffold count by 3.8% and increased the scaffold N50 by 376.7%. The curation process is described at https://gitlab.com/wtsi-grit/rapid-curation . PretextSnapshot was used to generate a Hi-C contact map of the final assembly.

### Assembly quality assessment

The Merqury.FK tool ( Rhie *et al.*, 2020) was run in a Singularity container ( Kurtzer *et al.*, 2017) to evaluate *k*-mer completeness and assembly quality for the primary and alternate haplotypes using the *k*-mer databases ( *k* = 31) computed prior to genome assembly. The analysis outputs included assembly QV scores and completeness statistics.

The genome was analysed using the BlobToolKit pipeline, a Nextflow implementation of the earlier Snakemake version ( Challis *et al.*, 2020). The pipeline aligns PacBio reads using minimap2 ( Li, 2018) and SAMtools ( Danecek *et al.*, 2021) to generate coverage tracks. It runs BUSCO ( Manni *et al.*, 2021) using lineages identified from the NCBI Taxonomy ( Schoch *et al.*, 2020). For the three domain-level lineages, BUSCO genes are aligned to the UniProt Reference Proteomes database ( Bateman *et al.*, 2023) using DIAMOND blastp ( Buchfink *et al.*, 2021). The genome is divided into chunks based on the density of BUSCO genes from the closest taxonomic lineage, and each chunk is aligned to the UniProt Reference Proteomes database with DIAMOND blastx. Sequences without hits are chunked using seqtk and aligned to the NT database with blastn ( Altschul *et al.*, 1990). The BlobToolKit suite consolidates all outputs into a blobdir for visualisation. The BlobToolKit pipeline was developed using nf-core tooling ( Ewels *et al.*, 2020) and MultiQC ( Ewels *et al.*, 2016), with containerisation through Docker ( Merkel, 2014) and Singularity ( Kurtzer *et al.*, 2017).

## Genome sequence report

### Sequence data

**Figure 2.**
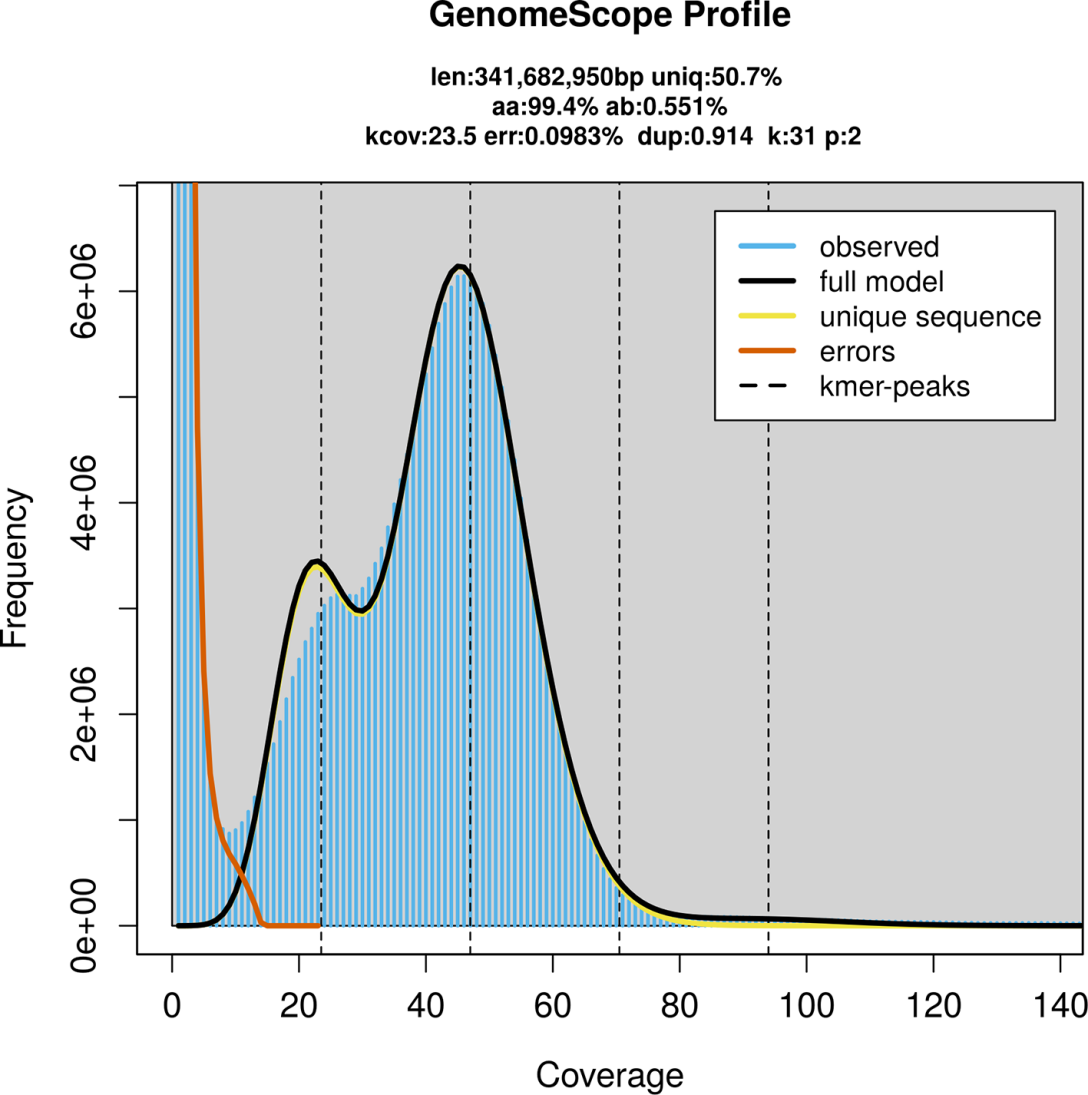
Frequency distribution of *k*-mers generated using GenomeScope2. The plot shows observed and modelled *k*-mer spectra, providing estimates of genome size, heterozygosity, and repeat content based on unassembled sequencing reads.

PacBio sequencing of the *Netelia inedita* specimen generated 16.60 Gb (gigabases) from 1.50 million reads, which were used to assemble the genome. GenomeScope2.0 analysis estimated the haploid genome size at 342.02 Mb, with a heterozygosity of 0.55% and repeat content of 49.34% ( Figure 2). These estimates guided expectations for the assembly. Based on the estimated genome size, the sequencing data provided approximately 47× coverage. Hi-C sequencing produced 122.43 Gb from 810.79 million reads, which were used to scaffold the assembly. RNA sequencing data were also generated and are available in public sequence repositories. Table 1 summarises the specimen and sequencing details.

**Table 1.** Specimen and sequencing data for BioProject PRJEB65668.

Platform	PacBio HiFi	Hi-C	RNA-seq
**ToLID**	iyNetThom1	iyNetThom1	iyNetThom1
**Specimen ID**	NHMUK014425675	NHMUK014425675	NHMUK014425675
**BioSample (source individual)**	SAMEA110044018	SAMEA110044018	SAMEA110044018
**BioSample (tissue)**	SAMEA14448787	SAMEA14448788	SAMEA14448789
**Tissue**	thorax	head	abdomen
**Instrument**	Sequel IIe	Illumina NovaSeq 6000	Illumina NovaSeq 6000
**Run accessions**	ERR12015733	ERR12035241	ERR12035242
**Read count total**	1.50 million	810.79 million	73.51 million
**Base count total**	16.60 Gb	122.43 Gb	11.10 Gb

### Assembly statistics

The primary haplotype was assembled, and contigs corresponding to an alternate haplotype were also deposited in INSDC databases. The final assembly has a total length of 403.61 Mb in 525 scaffolds, with 297 gaps, and a scaffold N50 of 36.33 Mb ( Table 2).

**Table 2.** Genome assembly statistics.

**Assembly name**	iyNetThom1.1
**Assembly accession**	GCA_964017225.1
**Alternate haplotype accession**	GCA_964017215.1
**Assembly level**	chromosome
**Span (Mb)**	403.61
**Number of chromosomes**	6
**Number of contigs**	822
**Contig N50**	1.39 Mb
**Number of scaffolds**	525
**Scaffold N50**	36.33 Mb
**Organelles**	Mitochondrion: 26.77 kb

**Figure 3.**
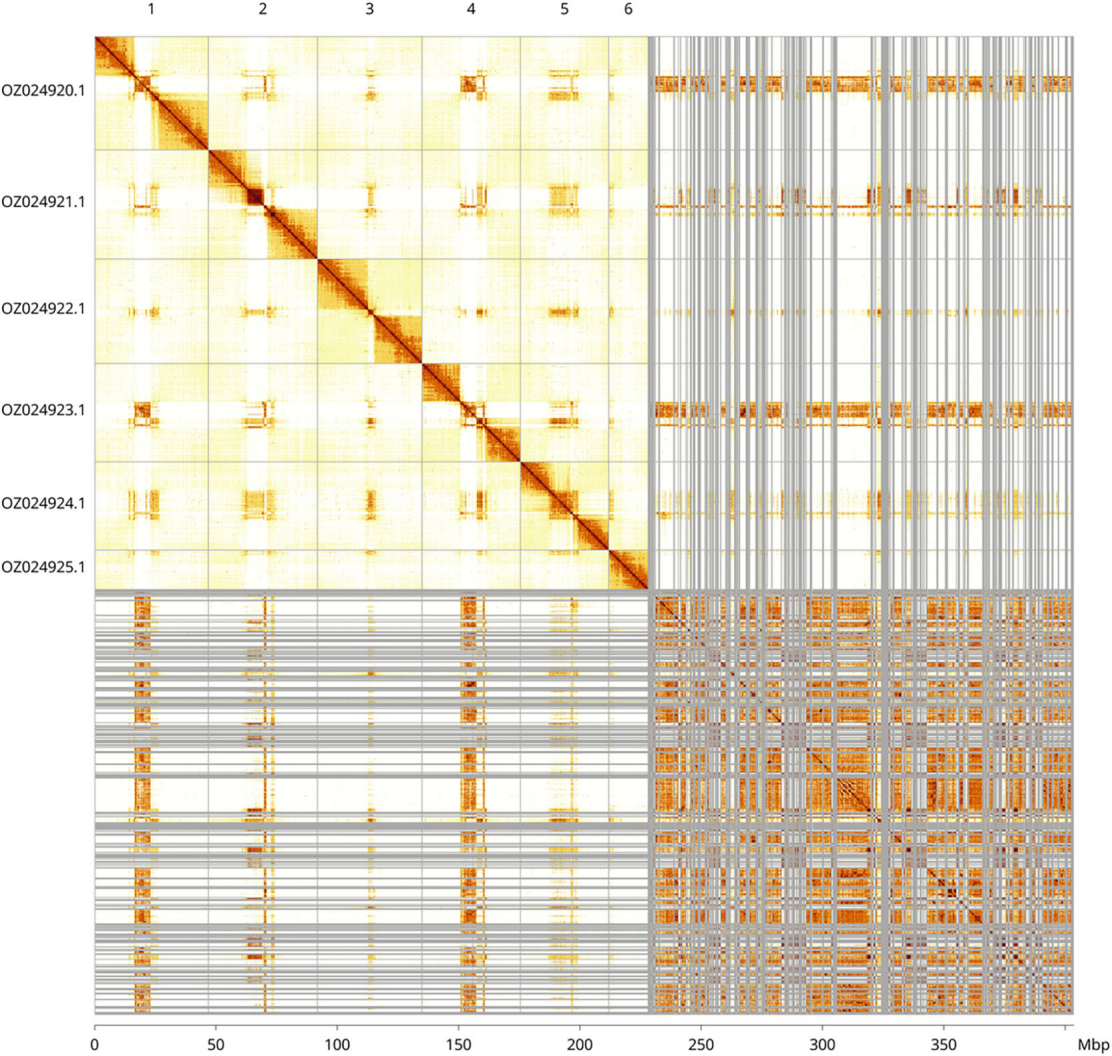
Hi-C contact map of the *Netelia inedita* genome assembly. Assembled chromosomes are shown in order of size and labelled along the axes, with a megabase scale shown below. The plot was generated using PretextSnapshot.

Most of the assembly sequence (56.53%) was assigned to 6 chromosomal-level scaffolds. These chromosome-level scaffolds, confirmed by Hi-C data, are named according to size ( Figure 3; Table 3). The exact order and orientation of the contigs on chromosome 4 (15.7–22.5 Mb) are unknown.

**Table 3.** Chromosomal pseudomolecules in the primary genome assembly of *Netelia inedita* iyNetThom1.

INSDC accession	Molecule	Length (Mb)	GC%
OZ024920.1	1	46.93	42
OZ024921.1	2	45.01	43.50
OZ024922.1	3	43.05	41
OZ024923.1	4	40.58	42.50
OZ024924.1	5	36.33	42
OZ024925.1	6	16.28	41

The mitochondrial genome was also assembled (length 26.77 kb, OZ024926.1). This sequence is included as a contig in the multifasta file of the genome submission and as a standalone record.

### Assembly quality metrics

**Figure 4.**
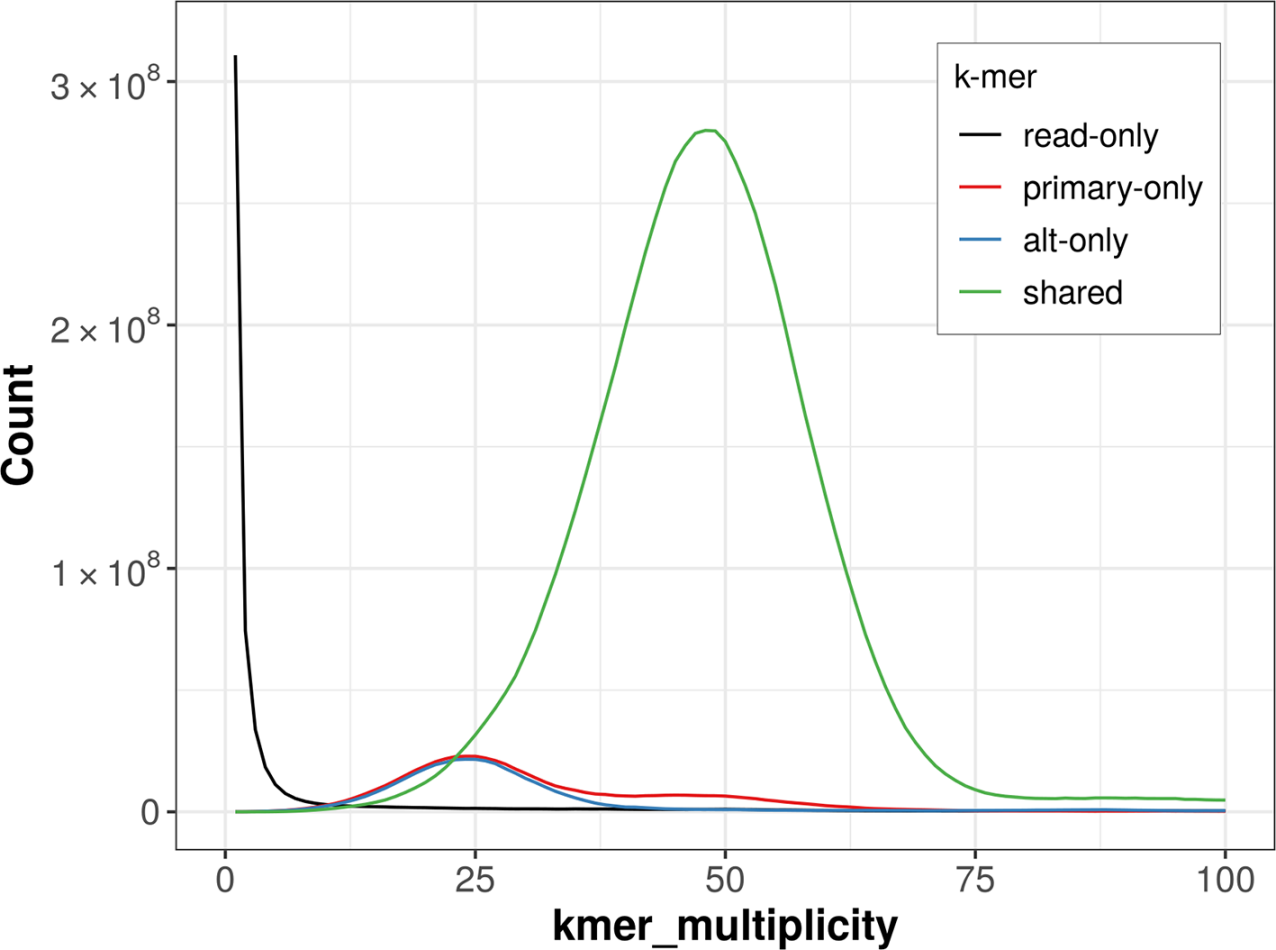
Evaluation of *k*-mer completeness using MerquryFK. This plot illustrates the recovery of *k*-mers from the original read data in the final assemblies. The horizontal axis represents *k*-mer multiplicity, and the vertical axis shows the number of *k*-mers. The black curve represents *k*-mers that appear in the reads but are not assembled. The green curve corresponds to *k*-mers shared by both haplotypes, and the red and blue curves show *k*-mers found only in one of the haplotypes.

The combined primary and alternate assemblies achieve an estimated QV of 58.3. The *k*-mer completeness is 90.33% for the primary assembly, 88.34% for the alternate haplotype, and 98.32% for the combined assemblies ( Figure 4).

**Figure 5.**
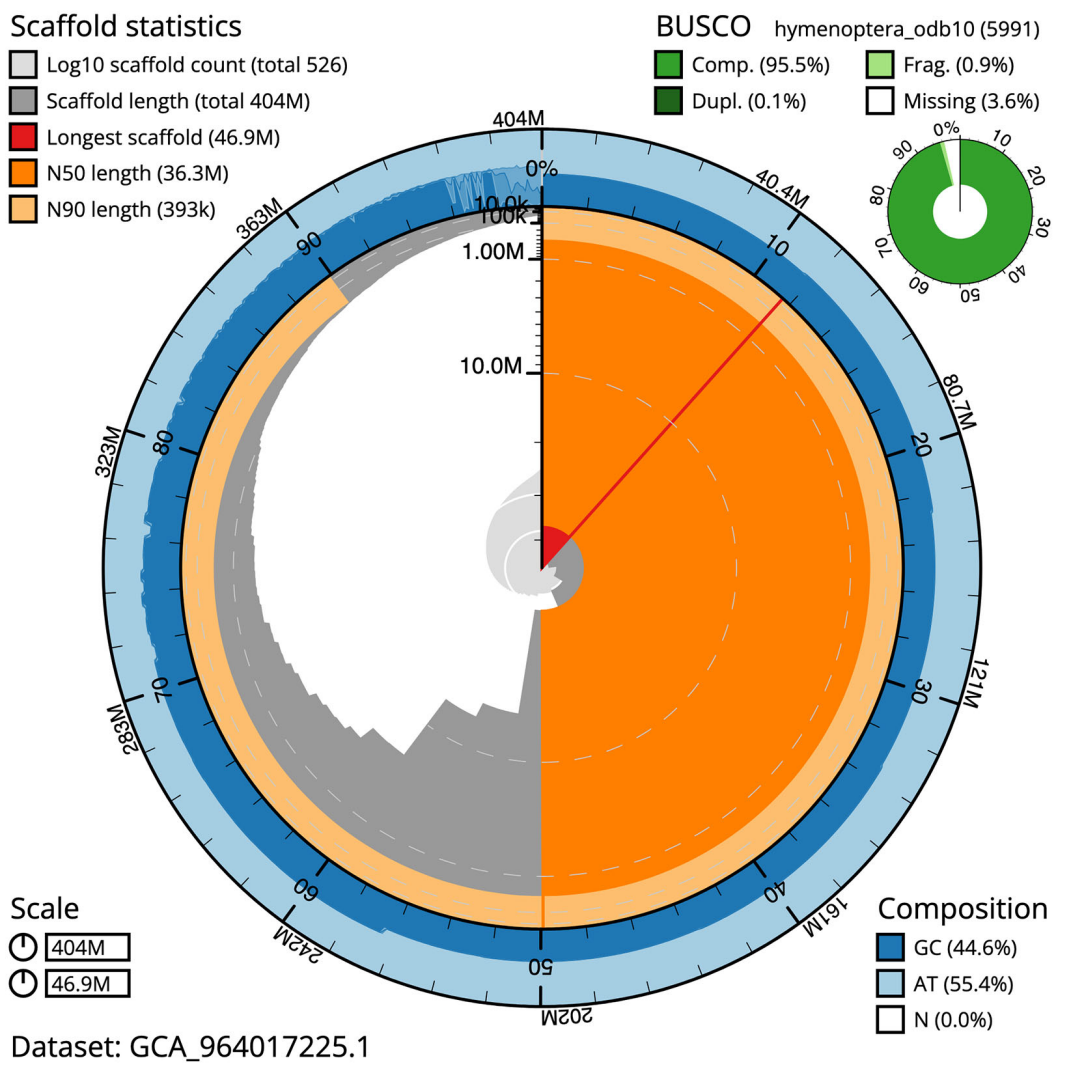
Assembly metrics for iyNetThom1.1. The BlobToolKit snail plot provides an overview of assembly metrics and BUSCO gene completeness. The circumference represents the length of the whole genome sequence, and the main plot is divided into 1 000 bins around the circumference. The outermost blue tracks display the distribution of GC, AT, and N percentages across the bins. Scaffolds are arranged clockwise from longest to shortest and are depicted in dark grey. The longest scaffold is indicated by the red arc, and the deeper orange and pale orange arcs represent the N50 and N90 lengths. A light grey spiral at the centre shows the cumulative scaffold count on a logarithmic scale. A summary of complete, fragmented, duplicated, and missing BUSCO genes in the endopterygota_odb10 set is presented at the top right. An interactive version of this figure can be accessed on the BlobToolKit viewer.

**Figure 6.**
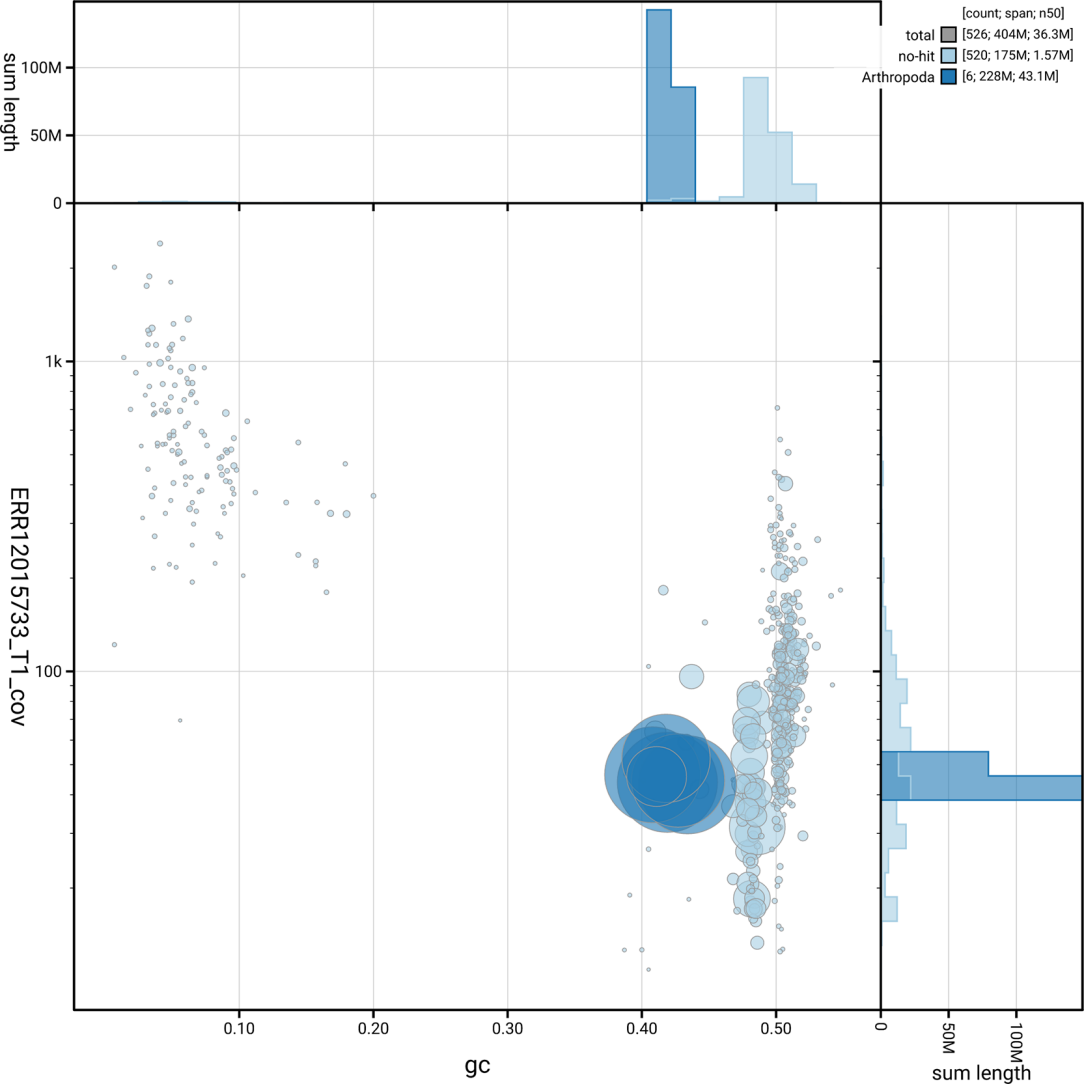
BlobToolKit blob plot for iyNetThom1.1. The plot shows base coverage (vertical axis) and GC content (horizontal axis). The circles represent scaffolds, with the size proportional to scaffold length and the colour representing phylum membership. The histograms along the axes display the total length of sequences distributed across different levels of coverage and GC content. An interactive version of this figure is available on the BlobToolKit viewer.

BUSCO v.5.5.0 analysis using the endopterygota_odb10 reference set ( *n* = 2 124) identified 99.1% of the expected gene set (single = 98.9%, duplicated = 0.2%). The snail plot in Figure 5 summarises the scaffold length distribution and other assembly statistics for the primary assembly. The blob plot in Figure 6 shows the distribution of scaffolds by GC proportion and coverage.

Table 4 lists the assembly metric benchmarks adapted from Rhie *et al.* (2021) and the Earth BioGenome Project Report on Assembly Standards September 2024. The EBP metric, calculated for the primary assembly, is **6.7.Q57**, meeting the recommended reference standard.

**Table 4.** Earth Biogenome Project summary metrics for the *Netelia inedita* assembly.

Measure	Value	Benchmark
EBP summary (primary)	6.7.Q57	6.C.Q40
Contig N50 length	1.39 Mb	≥ 1 Mb
Scaffold N50 length	36.33 Mb	= chromosome N50
Consensus quality (QV)	Primary: 57.6; alternate: 59.4; combined: 58.3	≥ 40
*k*-mer completeness	Primary: 90.33%; alternate: 88.34%; combined: 98.32%	≥ 95%
BUSCO	C:99.1% [S:98.9%, D:0.2%], F:0.2%, M:0.7%, n:2 124	S > 90%; D < 5%
Percentage of assembly assigned to chromosomes	56.53%	≥ 90%

**Notes:** The EBP summary uses log10(Contig N50); chromosome-level (C) or log10(Scaffold N50); Q (Merqury QV). BUSCO: C = complete; S = single-copy; D = duplicated; F = fragmented; M = missing; n = orthologues.

## Author information

Contributors are listed at the following links:

• Members of the Natural History Museum Genome Acquisition Lab
• Members of the Darwin Tree of Life Barcoding collective
• Members of the Wellcome Sanger Institute Tree of Life Management, Samples and Laboratory team
• Members of Wellcome Sanger Institute Scientific Operations – Sequencing Operations
• Members of the Wellcome Sanger Institute Tree of Life Core Informatics team
• Members of the Tree of Life Core Informatics collective
• Members of the Darwin Tree of Life Consortium

## Wellcome Sanger Institute – Legal and Governance

The materials that have contributed to this genome note have been supplied by a Darwin Tree of Life Partner. The submission of materials by a Darwin Tree of Life Partner is subject to the **‘Darwin Tree of Life Project Sampling Code of Practice’**, which can be found in full on the Darwin Tree of Life website. By agreeing with and signing up to the Sampling Code of Practice, the Darwin Tree of Life Partner agrees they will meet the legal and ethical requirements and standards set out within this document in respect of all samples acquired for, and supplied to, the Darwin Tree of Life Project. Further, the Wellcome Sanger Institute employs a process whereby due diligence is carried out proportionate to the nature of the materials themselves, and the circumstances under which they have been/are to be collected and provided for use. The purpose of this is to address and mitigate any potential legal and/or ethical implications of receipt and use of the materials as part of the research project, and to ensure that in doing so we align with best practice wherever possible. The overarching areas of consideration are:

• Ethical review of provenance and sourcing of the material
• Legality of collection, transfer and use (national and international)

Each transfer of samples is further undertaken according to a Research Collaboration Agreement or Material Transfer Agreement entered into by the Darwin Tree of Life Partner, Genome Research Limited (operating as the Wellcome Sanger Institute), and in some circumstances, other Darwin Tree of Life collaborators.

## Data Availability

European Nucleotide Archive: Netelia inedita. Accession number PRJEB65668. The genome sequence is released openly for reuse. The *Netelia inedita* genome sequencing initiative is part of the Darwin Tree of Life Project (PRJEB40665) and the Sanger Institute Tree of Life Programme (PRJEB43745). All raw sequence data and the assembly have been deposited in INSDC databases. The genome will be annotated using available RNA-Seq data and presented through the Ensembl pipeline at the European Bioinformatics Institute. Raw data and assembly accession identifiers are reported in Tables 1 and 2. Production code used in genome assembly at the WSI Tree of Life is available at https://github.com/sanger-tol . Table 5 lists software versions used in this study.
