# Supplementary material for: The genome sequence of a parasitoid wasp, Netelia inedita (Kokujev, 1899) (Hymenoptera: Ichneumonidae)
Source: Wellcome Open Res. 2026 Mar 18;11:174. [Version 1] doi: 10.12688/wellcomeopenres.26144.1 (PMC13049423; doi:10.12688/wellcomeopenres.26144.1)
Supplement: Peer review history

# Reviewer report: Reviewer response for version 1

**DOI:** 10.21956/wellcomeopenres.28791.r151174

**Published:** 2026-04-03

**Authors:** Željko Tomanović

**Affiliations:** 1 Serbia University of Belgrade, Belgrade, Serbia

The author presents the genome assembly of the Darwin wasp, Netelia inedita (Kokujev, 1899) (Tryphoninae), including the mitochondrial genome. A comprehensive and informative background on the taxonomy, biology, and ecology of this species is provided. The distinction between N. inedita and N. thomsonii Brauns, 1889, along with the author's clarification of the confusion in the literature regarding these two valid species and their synonymisation, is particularly significant. The collection and sequencing methodology is described in detail, and all analyses are conducted in accordance with the highest standards.

Are sufficient details of methods and materials provided to allow replication by others?

Yes

Is the rationale for creating the dataset(s) clearly described?

Yes

Are the datasets clearly presented in a useable and accessible format?

Yes

Are the protocols appropriate and is the work technically sound?

Yes

Reviewer Expertise:

parasitoid phylogeny

I confirm that I have read this submission and believe that I have an appropriate level of expertise to confirm that it is of an acceptable scientific standard.

# Reviewer report: Reviewer response for version 1

**DOI:** 10.21956/wellcomeopenres.28791.r151179

**Published:** 2026-04-02

**Authors:** Vahap Eldem

**Affiliations:** 1 Biology, Istanbul University, Istanbul, Turkey

The authors reported a chromosome level genome assembly of Netellia inedita, together with the associated sequencing datasets and assembly related analyses. The manuscript is generally suitable for a data note and reports the main assembly characteristics and supporting resources. However, several methodological and reporting points would benefit from further clarification.

 1. There is a discrepancy between the figure and the text regarding the BUSCO result. The figure reports 95.5%, whereas the text reports 99.1%. It is possible that different databases were used, for example Hymenoptera odb10 for the figure and Endopterygota odb10 for the text. However, the caption of Figure 5 also refers to Endopterygota odb10. The authors should clarify which BUSCO database was actually used and ensure consistency between the figure, caption, and main text.

 2. The chromosome percentage of assembly is very low (56.53%) compared to the benchmark (>90), and in that regard it seems far from meeting the standard. Is this expected in a Hi-C supported assembly?

 3. The read quality metrics for the sequencing data would be a good addition to Table 1, as they would help show the overall quality summary of the reads.

 4. For taxonomic identification, it is stated that DNA barcoding was applied. It would be helpful to add the accession number and a BLAST/BOLD result table to better show these results.

 5. The authors mentioned an RNA extraction and sequencing, yet the manuscript does not clearly state how these RNA data were used in the study. Maybe this can be clarified if it was included only for data generation for further downstream applications, such as supporting annotation etc.

Are sufficient details of methods and materials provided to allow replication by others?

Yes

Is the rationale for creating the dataset(s) clearly described?

Yes

Are the datasets clearly presented in a useable and accessible format?

Yes

Are the protocols appropriate and is the work technically sound?

Yes

Reviewer Expertise:

genomics, bioinformatics

I confirm that I have read this submission and believe that I have an appropriate level of expertise to confirm that it is of an acceptable scientific standard, however I have significant reservations, as outlined above.

# Reviewer report: Reviewer response for version 1

**DOI:** 10.21956/wellcomeopenres.28791.r151180

**Published:** 2026-03-23

**Authors:** Alexandra Cerqueira De Araujo

**Affiliations:** 1 University College London, London, UK

The authors present here a chromosome-level *de novo* assembly of the genome of *Netelia inedita *(Ichneumonidae, Hymenoptera). This is the first assembly available for this species and for the subgenus Paropheltes, which contains four species on the NCBI taxonomy database. This assembly is composed of 6 chromosome-level pseudomolecules and one mitochondrial genome. The assembly has a total length of 403.61 Mb and a N50 of 36.33 Mb. This assembly achieves a EBP reference standard of 6.7.Q57 and contains most of the orthologous genes found in hymenopterans (the assembly contains 95.5% of the complete BUSCO sequences according to Figure 5). All methods used to produce this data is reported in this manuscript and the code is available here https://github.com/sanger-tol.

 Minor comments:

 1- in "Assembly statistics"

 For the BUSCOs identified, are they mostly located on the assembled chromosomes? Which proportion of the BUSCOs identified can be found on those chromosome sequences?

 2- Regarding the species name and the NCBI/ENA referencing, the authors specify “ *Paniscus ineditus* Kokujev, 1899, is listed as a junior synonym of *Netelia thomsonii* (Brauns, 1889) in most sources, following Tolkanitz (1974) and Kasparyan & Tolkanitz (1999). However, *Netelia inedita* and *N. thomsonii* are clearly separate species, as recognised by Delrio (1974). Broad *et al.* (in prep.), in a review of European *Netelia*, separate the two species.”. However, on NCBI and ENA (EMBL-EBI), bioprojects and biosamples contain “Netelia thomsonii” in their title and description (20/03/2026). Although the organism section still specifies “Netelia inedita” and the assembly and SRAs associated to the species appear when looking specifically for *Netelia inedita*, I would contact NCBI and ENA to operate title and description changes to avoid confusions.

Are sufficient details of methods and materials provided to allow replication by others?

Yes

Is the rationale for creating the dataset(s) clearly described?

Yes

Are the datasets clearly presented in a useable and accessible format?

Yes

Are the protocols appropriate and is the work technically sound?

Yes

Reviewer Expertise:

genomics

I confirm that I have read this submission and believe that I have an appropriate level of expertise to confirm that it is of an acceptable scientific standard.
